# Biopolymer Surface Modification as a Strategy for Conferring “Stealth-like” Characteristics of Xanthohumol-Loaded Liposomes

**DOI:** 10.3390/polym18141724

**Published:** 2026-07-13

**Authors:** Plamen Simeonov, Velislava Todorova, Tsvetelina Batsalova, Balik Dzhambazov, Stanislava Ivanova, Plamen Katsarov

**Affiliations:** 1 Department of Pharmaceutical Technology and Biopharmacy, Faculty of Pharmacy, Medical University of Plovdiv, 15A Vasil Aprilov Blvd, 4002 Plovdiv, Bulgaria; plamen.simeonov@mu-plovdiv.bg; 2 Research Institute at Medical University of Plovdiv (RIMU), 4002 Plovdiv, Bulgaria; velislava.todorova@mu-plovdiv.bg (V.T.); stanislava.ivanova@mu-plovdiv.bg (S.I.); 3 Department of Pharmacognosy and Pharmaceutical Chemistry, Faculty of Pharmacy, Medical University of Plovdiv, 15A Vasil Aprilov Blvd, 4002 Plovdiv, Bulgaria; 4 Department of Developmental Biology, Faculty of Biology, Paisii Hilendarski University of Plovdiv, 24 Tsar Assen Str., 4000 Plovdiv, Bulgaria; tsvetelina@uni-plovdiv.bg (T.B.); balik@uni-plovdiv.bg (B.D.)

**Keywords:** xanthohumol, liposomes, iota-carrageenan, fucoidan, ionotropic cross-linking, surface modification, stealth nanocarriers, macrophage uptake, controlled release

## Abstract

Xanthohumol (XN), a prenylated chalcone isolated from *Humulus lupulus* L., exhibits a wide range of biological activities, including antioxidant, anti-inflammatory, and chemopreventive effects. However, its therapeutic application is limited by poor aqueous solubility, low chemical stability, and rapid clearance from the systemic circulation. The present study aimed to develop and characterize a novel nano-sized drug-delivery system for XN that combines favourable colloidal stability, efficient encapsulation, sustained release, and reduced recognition by macrophages (“stealth-like” properties). To achieve this, XN-loaded cationic liposomes were coated with two marine polysaccharides, iota-carrageenan (CAR) and fucoidan (FUC), followed by Ca^2+^-mediated cross-linking. Liposomes were prepared by the ethanol injection method, and formulation parameters were optimized using a 2^3^ + 1 full factorial design. Surface modification and cross-linking conditions were further optimized through polyelectrolyte titration and a Taguchi L9 orthogonal array. The resulting nanocarriers were evaluated for particle size, polydispersity, ζ-potential, encapsulation efficiency, release behavior, and cellular uptake. Both coatings significantly prolonged XN release compared with uncoated liposomes, with CAR-coated vesicles providing the most sustained release (≈55% over 48 h). In RAW264.7 macrophages, 50 µg/mL CAR-coated liposomes reduced cellular uptake by approximately 74% following 1-h incubation relative to uncoated controls and maintained this reduction over 2 h whereas FUC-coated vesicles afforded only transient early evasion. The cross-linked iota-carrageenan coating thus represents a promising strategy for conferring stable “stealth-like” characteristics to XN-loaded liposomes intended for prolonged drug delivery.

## 1. Introduction

**Figure 1:**
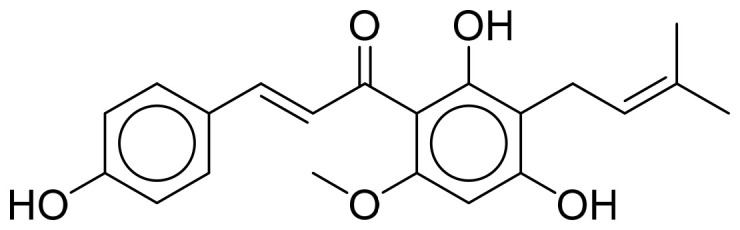
Chemical structure of xanthohumol, a prenylated chalcone (drawn using ChemDraw, version 25.0.2.14, PerkinElmer).

Hops (*Humulus lupulus* L., Cannabaceae) have long been valued not only as a key ingredient in the brewing industry but also as a source of biologically active secondary metabolites. Among the prenylflavonoids isolated from the female inflorescences of hops, xanthohumol (XN, Figure 1)—a prenylated chalcone—has emerged as one of the most pharmacologically promising compounds [1]. Over the last two decades, XN has been reported to display a remarkably broad spectrum of bioactivities, including antioxidant, anti-inflammatory, antimicrobial, antiviral, hepatoprotective, antidiabetic, cardioprotective, and chemopreventive effects [2]. Its anticancer activity has attracted particular attention, with studies demonstrating apoptosis induction, inhibition of angiogenesis, modulation of NF-κB and Wnt/β-catenin signalling, and suppression of cytochrome P450-mediated procarcinogen activation across a variety of tumour cell lines [3,4].

Despite this attractive pharmacological profile, the translation of XN into clinically or nutraceutically relevant formulations is limited by its unfavourable physicochemical properties. XN is a highly lipophilic molecule (logP ≈ 4.5, where logP is the logarithm of the n-octanol/water partition coefficient) with low aqueous solubility (<1 µg/mL), poor oral bioavailability, and substantial chemical instability—undergoing rapid intramolecular cyclisation to its less active flavanone isomer, isoxanthohumol, under acidic, thermal, or light-exposed conditions [5,6]. These limitations impose significant constraints on the in vivo plasma concentrations, thereby impeding the development of effective dosage forms. Consequently, considerable effort has been directed towards the design of advanced delivery systems capable of enhancing the solubility, stability, and bioavailability of XN, including cyclodextrin inclusion complexes, micelles, solid lipid nanoparticles, polymeric nanoparticles, and lipid-based vesicular carriers [7,8].

Among these strategies, liposomes—spherical vesicles composed of one or more phospholipid bilayers enclosing an aqueous core—represent one of the most extensively investigated and clinically validated nanocarriers [9]. Their structural similarity to biological membranes endows them with excellent biocompatibility and biodegradability, while their amphiphilic architecture enables the simultaneous encapsulation of hydrophilic compounds within the inner aqueous compartment and of lipophilic compounds, such as XN, within the phospholipid bilayer [10]. Liposomal encapsulation has been shown to protect XN from oxidative and photochemical degradation, increase its water solubility, and improve its cellular uptake [11]. Nevertheless, conventional liposomes still suffer from several well-documented drawbacks, including physical instability during storage (aggregation, fusion, and phospholipid hydrolysis), drug leakage, burst release, susceptibility to bile salts and digestive enzymes in the gastrointestinal tract, and rapid clearance by the mononuclear phagocyte system following systemic administration [12]. These limitations have motivated extensive research into surface engineering of liposomes to improve their robustness and modulate their biological fate.

Coating liposomes with biocompatible polysaccharides has emerged as a particularly attractive surface-modification strategy. Natural polysaccharides such as chitosan, alginate, pectin, hyaluronic acid, carboxymethyl cellulose, and various gums are inexpensive, non-toxic, biodegradable materials and bear functional groups (–OH, –NH_2_, –COOH) that allow versatile chemical and physical modification [13]. Adsorption of polyelectrolyte chains onto the liposomal surface—typically driven by electrostatic and hydrogen-bonding interactions—produces a hydrated polymeric corona that sterically and/or electrostatically stabilises the vesicles, suppresses phospholipid oxidation, hinders premature drug leakage, and confers mucoadhesive properties beneficial for oral, buccal, nasal, and ocular delivery [14,15]. Polysaccharide coatings can additionally provide pH- and enzyme-responsive behaviour, enabling site-specific release in the gastrointestinal tract or in the tumour microenvironment [16,17].

The performance of polysaccharide-coated liposomes can be further reinforced through chemical or ionic cross-linking of the surface polymer layer [18,19]. Cross-linking—for example, ionotropic gelation of alginate or pectin with Ca^2+^, electrostatic cross-linking of chitosan with sodium tripolyphosphate, or covalent cross-linking with naturally derived agents such as genipin—converts the loosely adsorbed polymer corona into a denser, network-like shell [19,20]. This dense shell can improve vesicle integrity against mechanical stress, dilution, freeze-drying, and gastrointestinal fluids, while simultaneously enabling sustained and more controllable release kinetics of the encapsulated drug load [18,21]. Cross-linked polysaccharide-coated liposomes thus combine the encapsulation versatility of liposomes with the mechanical and biological resilience of hydrogel-like polymeric shells, representing a particularly suitable platform for the delivery of lipophilic molecules sensitive to stability, such as XN.

Despite the clear rationale, reports describing the encapsulation of XN into cross-linked polysaccharide-coated liposomes remain limited, and a systematic understanding of how the coating composition and cross-linking conditions influence the physicochemical stability and release performance of XN-loaded vesicles is still lacking. Thus, the aim of the present study was to develop and characterize XN-loaded liposomes coated with iota-carrageenan and fucoidan and subsequently cross-linked with CaCl_2_, and to evaluate the resulting nanocarriers in terms of particle size, zeta potential, polydispersity index, encapsulation efficiency, in vitro release behaviour, and test whether the constructed shell will successfully provide a shield against macrophage uptake, giving potential “stealth-like” properties of the formulated vesicles.

## 2. Materials and Methods

### 2.1. Chemicals and Reagents

Xanthohumol (Mw 354.39 g/mol) was purchased from Cayman Chemicals (Ann Arbor, MI, USA), LIPOID PC 16:0/16:0 (1,2-Dipalmitoyl-sn-glycero-3-phosphocholine, DPPC), and LIPOID DOTAP-Cl (1,2-Dioleoyloxy-3-trimethylammonium-propane chloride, DOTAP) were purchased from Lipoid GmbH (Ludwigshafen, Germany). Cholesterol (from sheep wool, ≥92.5%), Fucoidan (from Fucus vesiculosus ≥ 95%), Carrageenan (iota-type), CaCl_2_ (Mw: 110.98 g/mol, ≥97.0%), FITC (Fluorescein 5(6)-isothiocyanate, Mw 389.38) and DAPI (4′,6-Diamidino-2-phenylindole dihydrochloride) were purchased from Sigma-Aldrich (St. Louis, MO, USA). Trisodium citrate was purchased from Fillab (Plovdiv, Bulgaria). Ethanol (99.9%) was purchased from RaiChim (Plovdiv, Bulgaria), and acetonitrile and methanol (analytical grade) were purchased from Merck KGaA (Darmstadt, Germany).

### 2.2. Preparation of Xanthohumol-Loaded Liposomes

Xanthohumol-loaded liposomes were prepared by the ethanol injection method, described previously [22]. Briefly, DPPC, DOTAP, cholesterol, and Xanthohumol were dissolved in 10 mL of absolute ethanol. The resulting solution was heated above the phase transition temperature of the lipids (45 °C) and injected rapidly through 27G needle into 100 mL of ultrapure water, which was also heated to the same temperature. The suspension was kept under magnetic stirring for a period of 1 h, and then the residual ethanol was evaporated using the BUCHI RII Rotavapor rotary vacuum evaporator (BÜCHI Labortechnik AG, Flawil, Switzerland) under reduced pressure. After that, the liposomal suspension was stored at 4 °C overnight to allow complete vesicle formation. Nine models were prepared with varying lipid concentration, DPPC: DOTAP: Cholesterol ratio, and Lipid phase: Xanthohumol ratio using 2^3^ + 1 full factorial experimental matrix. All preparations were performed as three independent batches to ensure reproducibility.

### 2.3. Surface Modification of the Liposomes

The formulated xanthohumol-loaded liposomes were further modified by the method of single-layer deposition with subsequent cross-linking of the formed coating with CaCl_2_ [23]. Two anionic marine polysaccharides were selected, namely iota-carrageenan and fucoidan. The optimal polysaccharide-to-liposome ratio for single-layer deposition was determined by polyelectrolyte titration based on ζ-potential measurements. A series of mixtures was prepared at different polysaccharide-to-liposome weight ratios (*w*/*w*): (1:15, 1:10, 1:5, 1:3, 1:1, 3:1, 5:1, 10:1, 15:1). For each ratio, the defined volume of liposomes with a concentration of 1.0 mg/mL was added dropwise to a solution of polysaccharide with a concentration of 1.0 mg/mL under continuous magnetic stirring. The mixtures were incubated for 60 min at room temperature. The ζ-potential of each mixture was measured, and the optimal ratio for each polysaccharide was defined as the point at which electrostatic saturation of the liposomal surface was reached (i.e., the ratio beyond which no further significant change in ζ-potential occurred).

After defining the optimal polysaccharide-to-liposome ratios, the liposomes were coated with a single layer of the corresponding polysaccharide. Nine models were prepared using an L9 Taguchi orthogonal array. A defined amount of liposomes at concentration of 1 mg/mL was added dropwise to a solution of polysaccharide with varying concentrations (0.5 mg/mL, 1.0 mg/mL, 2.0 mg/mL) at a fixed polysaccharide: liposomes ratio. The suspension was incubated for 30 min, 60 min, or 120 min. Subsequently, a solution of CaCl_2_ was added at varying concentrations (1 mM, 5 mM, 10 mM), and the suspension was kept stirring for another 60 min to achieve complete equilibrium.

### 2.4. Liposomes Particle Average Size, Particle Size Distribution and ζ-Potential

The average particle diameter, particle size distribution, and ζ-potential of the formulated liposomes as well as the surface-modified liposomes were determined via dynamic and electrophoretic light scattering with a particle size analyser (Zetasizer UltraRed, Malvern Panalytical Ltd., Malvern, UK).

### 2.5. HPLC-PDA Quantitative Analysis of Xanthohumol

#### 2.5.1. Preparation of Standard and Test Solutions and Chromatographic Conditions

The standard compound XN was prepared as a stock solution at a concentration of 1 mg/mL using an acetonitrile/water mixture (50:50, *v*/*v*). To enhance dissolution, the solution was treated in an ultrasonic bath (Bandelin, Berlin, Germany). Subsequently, working standard solutions were obtained by serial dilution with the same acetonitrile/water mixture to cover the required validation range. Test samples were similarly diluted with acetonitrile/water (50:50, *v*/*v*) to fall within the validated concentration range. The method was established using an LC40-PDA system (Shimadzu, Kyoto, Japan) fitted with a Shim-pack C18 column (150 mm × 4.6 mm, 3 μm, Shimadzu, Kyoto, Japan). Isocratic elution was applied to improve separation efficiency. The mobile phase consisted of water (A), methanol (B), and acetonitrile (C), with an overall composition of 25% A, 10% B, and 65% C. The flow rate was maintained at 0.6 mL/min, and the column temperature was set to 40 °C. A sample volume of 10 μL was injected for analysis. UV–Vis spectra were recorded over the range of 190–800 nm, while chromatographic detection was performed at 370 nm. Data acquisition and processing were carried out using LabSolutions software (version 5.118, Shimadzu, Kyoto, Japan). All test samples for entrapment efficiency and in vitro release determination were diluted as required so that their xanthohumol concentrations fell within the validated calibration range.

#### 2.5.2. Validation of the HPLC-PDA Method

The developed method was validated in accordance with the guidelines of the International Council for Harmonisation of Technical Requirements for Pharmaceuticals for Human Use (EMA ICH Q2(R2) Validation of Analytical Procedures—Scientific Guideline) [24]. For Linearity, quantitative analysis was performed using the external standard method. Calibration standards were prepared at six concentration levels within the range of 5–75 µg/mL and analyzed in triplicate to establish calibration curves. The linearity of the method was evaluated by plotting the peak area versus the nominal concentration of the standards. The coefficient of determination (R^2^) was calculated as an indicator of linearity. The parameters Limit of detection (LD) and quantification (LQ) were calculated based on the linearity data, using the standard deviation of the response and the slope of the calibration curve. The accuracy of the proposed method was assessed by determining the percentage recovery. Three quality control levels were examined for each analyte: low (10 µg/mL), medium (30 µg/mL), and high (50 µg/mL). The recovery results demonstrated that the method provides satisfactory accuracy across the investigated concentration range. Method precision was evaluated in terms of both intra-day and inter-day repeatability. Intra-day precision was determined by analyzing freshly prepared samples at the three quality control levels, each in six replicates within a single day. Inter-day precision was assessed by analyzing freshly prepared samples at the same concentration levels over three consecutive days, with six replicates per level. The parameter robustness was evaluated by examining the effect of variations in column temperature on chromatographic performance. The column temperature was deliberately varied between 38 °C and 42 °C, and the retention times of the analytes were monitored. No significant changes in resolution were observed, indicating that small temperature fluctuations did not adversely affect the chromatographic separation, thereby confirming the robustness of the method. In addition, the influence of the flow rate was also investigated by applying a variation of ±2% from the nominal value. These minor changes did not produce any significant effect on the chromatographic performance, further demonstrating the stability and reliability of the developed method.

The results for linearity, as well as the calculated limits of detection and quantification, are summarized in Table S1, whereas the outcomes of the accuracy and precision assessments are presented in Tables S2 and S3, respectively, presented as Supplementary Materials.

### 2.6. Entrapment Efficiency of Xanthohumol (EE)

The EE was determined for the unmodified liposomes as well as the modified models by measurement of the incorporated xanthohumol. Unentrapped xanthohumol was separated by ultracentrifugation [25]. An aliquot of each formulation was centrifuged at 30,070× *g* for 90 min at 4 °C using a Sigma 3-18KS centrifuge equipped with a fixed-angle rotor (Sigma Laborzentrifugen GmbH, Osterode am Harz, Germany). The pellet containing the vesicles was incubated with methanol and sonicated for 10 min to ensure complete lysis of the vesicles and complete solubilization of xanthohumol. For the surface-modified liposomes, the same procedure was applied, but prior to incubation with methanol, 50 mM trisodium citrate was added to chelate Ca^2+^ and incubated for 30 min, in order to dissolve the coating shell. Then, the liposomes were subjected to ultracentrifugation at the aforementioned conditions, and then the pellet was incubated with methanol to solubilize the xanthohumol. The EE was calculated according to the following Equation (1):

*EE*% = *W_entrapped_*/*W_total_* × 100 (1)

where:

W*_entrapped_*—amount of incorporated Xanthohumol

W*_total_*—total amount of Xanthohumol

For surface-modified liposomes, W*_total_* was taken as the mass of xanthohumol entrapped in the optimal uncoated formulation prior to the coating step.

### 2.7. In Vitro Drug Release Study

The in vitro release of xanthohumol from the formulated uncoated and coated liposome models was evaluated using the dialysis bag method. The release medium consisted of phosphate-buffered saline (PBS, pH 7.4) supplemented with 20% (*v*/*v*) methanol to enhance solubilization of the poorly water-soluble xanthohumol [3,26,27]. The release medium was freshly prepared on the day of the experiment and equilibrated at 37 ± 0.5 °C prior to use. An aliquot of each formulation was placed inside a pre-soaked, 12 kDa MWCO dialysis membrane (Sigma, MWCO 12 kDa), which was hydrated in ultra-pure water overnight and sealed with a plastic clamp. Each bag was then immersed in a beaker containing 200 mL of release medium and maintained under constant gentle agitation on an electromagnetic stirrer at 150 rpm and 37.0 ± 0.5 °C. Aliquots of 2 mL were withdrawn at set intervals and replaced with fresh medium. The amount of released xanthohumol was determined by the method described in Section 2.5.

To confirm that the dialysis membrane did not retain xanthohumol and bias the measured release, a control experiment was performed in which 10 mL of free (non-encapsulated) xanthohumol solution, prepared in the release medium, was placed in the dialysis bag and dialysed against 200 mL of the same medium under conditions identical to the release experiments. After 1 h, approximately 97% of the xanthohumol was recovered in the acceptor compartment, and approximately 2.8% remained in the donor compartment, giving a mass balance of ~99.8%. The near-complete recovery confirms that the membrane was freely permeable to xanthohumol and did not significantly adsorb or retain the drug.

### 2.8. In Vitro Macrophage Uptake Assay

#### 2.8.1. Preparation of FITC-Labelled Liposomes

For cellular uptake studies, fluorescently labelled liposomes were prepared with the same lipid composition, coating, and cross-linking as the xanthohumol-loaded formulations, with fluorescein isothiocyanate (FITC) encapsulated within the aqueous core as a fluorescent tracer. FITC was added to the aqueous hydration phase at an initial concentration of 0.1 mg/mL. The liposomes were formed by the ethanol injection method (Section 2.2) and coated and cross-linked as described (Section 2.3). Unencapsulated free FITC was removed prior to all cellular experiments by ultracentrifugation under the same conditions used for the xanthohumol-loaded formulations (30,070× *g* for 90 min at 4 °C), and the amount of FITC incorporated was determined subsequently to be 0.052 mg/mL, corresponding to an encapsulation efficiency of approximately 52%. These FITC-labelled liposomes, matched in composition and surface modification to the drug-loaded formulations, were used to assess cellular uptake.

#### 2.8.2. Cell Lines and Culture Conditions

The following cell lines were used in the experiments: A549 (ATCC CCL-185™)—human lung adenocarcinoma cells, HFFC—human foreskin fibroblasts (CLS Cell Lines Service GmbH, Eppelheim, Germany), RAW264.7 (ATCC TIB-71)—a mouse macrophage cell line established from a tumor induced by the Abelson leukemia virus. The carcinoma cell line and RAW264.7 macrophages were cultured in Dulbecco’s Modified Eagle Medium (DMEM) (Merck KGaA, Darmstadt, Germany), supplemented with 10% fetal bovine serum (FBS) (Merck KGaA, Darmstadt, Germany) and a mixture of antibiotics (100 µg/mL streptomycin and 100 IU penicillin /Merck KGaA, Darmstadt, Germany/). HFFC cells were cultured in DMEM with 15% FCS and antibiotics (100 µg/mL streptomycin and 100 IU penicillin /Merck KGaA, Darmstadt, Germany/). The cells were cultured at 37 °C in an incubator maintaining high humidity and a gas mixture consisting of 5% CO_2_ and 95% atmospheric air.

#### 2.8.3. In Vitro Cell Uptake Assay

Upon reaching 80% confluency, RAW264.7, A549, and HFFC cells were detached from the culture dish, and the concentration of viable cells in the resulting cell suspensions was determined. Next, a cell suspension with a concentration of 2 × 10^5^ cells/mL was prepared and seeded onto a 96-well culture plate (TPP, Trasadingen, Switzerland) at a volume of 200 µL per well. After 24 h, RAW264.7 cells were stimulated with lipopolysaccharide (LPS) solution, which was added to the culture medium to a final concentration of 1 μg/mL. The cells were cultured in LPS-containing medium for 24 h. Subsequently, the cells were washed with DPBS and treated with fluorescein isothiocyanate (FITC)-labelled liposomes for 1 and 2 h. A549 and HFFC cells were seeded onto a 96-well culture plate (TPP, Trasadingen, Switzerland) at a concentration of 2 × 10^5^ cells/mL, and after 24 h, the cells were treated with 50 µg/mL fluorescein isothiocyanate (FITC)-labelled liposomes for 1 and 2 h. At the end of the incubation period, all cell types were washed three times with DPBS, and liposome uptake by the cells was analyzed based on fluorescence signal detection (excitation 495 nm/emission 525 nm) using a SpectraMax i3x spectrophotometer (Molecular Devices, San Jose, CA, USA).

To determine the amount of fluorescent dye released from the liposomes during cell culture, the culture medium was collected after incubating the cells with FITC-labeled liposomes for 1 and 2 h. The collected medium was then centrifuged at 13,400 rpm for 1 h to pellet intact liposomes and cellular debris, and the resulting supernatant was analysed. The concentration of FITC in the resulting supernatant was assessed using a SpectraMax i3x spectrophotometer (Molecular Devices, San Jose, CA, USA) and a reference FITC standard (FITC in two-fold serial dilution in cell culture medium in the concentration range 0.00625–100 µg/mL). The results demonstrated that the concentration of fluorescent dye released from the liposomes following 1-h and 2-h incubation with LPS-stimulated RAW264.7 cells ranged from 0.010 to 0.035 µg/mL.

For analysis by fluorescence microscopy, the method described by Wang et al. [28] was used with some modifications. RAW264.7 cells were seeded onto sterile slides at a concentration of 2 × 10^5^ cells/mL, incubated for 24 h, and then stimulated with 1 μg/mL LPS for 24 h. Subsequently, the cells were washed with DPBS and treated with FITC-loaded liposomes for 2 h. Afterward, the cells were washed three times with DPBS and fixed with a 4% paraformaldehyde solution for 20 min at room temperature. After fixation, the cells were washed twice with DPBS and incubated in the dark with a 0.75 µg/mL solution of DAPI for 10 min at room temperature. The cells were then washed again with DPBS and observed using a Leica DM1000 LED epifluorescence microscope equipped with an I3 filter Leica Microsystems (Wetzlar, Germany) and a camera (Leica DM 2000 LED, Leica Microsystems, Wetzlar, Germany), using Leica Application Suite (LAS X5.0.2) software to document and analyze the images obtained.

#### 2.8.4. Cell Viability Evaluations

To assess the viability of cells treated for 1 and 2 h with FITC-labelled liposomes, MTT assays were performed. A549 and HFFC cells were seeded and treated with liposomes as described in the previous subsection. RAW264.7 cells were seeded, stimulated with LPS, and treated with liposomes as described in the previous subsection. At the end of the 1- and 2-h incubation periods, the cells were washed three times with DPBS and incubated at 37 °C in cell culture medium containing 0.5 mg/mL 3-(4,5-dimethylthiazol-2-yl)-2,4-diphenyltetrazolium bromide (MTT) solution (Merck KGaA, Germany) for 2 h. During this incubation period, the yellow tetrazolium salt MTT is reduced by viable cells in the culture to purple formazan. Then, the culture medium was removed, and the formazan accumulated in cells was dissolved using 100 µL/well of DMSO. The culture plates were incubated for 15 min at room temperature on a shaker. After that, absorbance at 570 nm was measured using a Synergy-2 reader (BioTek, USA). The cytotoxic agent mitomycin C served as a positive control for all cell viability tests. All samples were analysed in triplicate. Results were expressed as the percentage of cell viability, calculated relative to the absorbance values of untreated control cells cultured for the same duration under standard growth conditions.

### 2.9. Statistical Analysis

Statistical analysis of the factorial design, as well as the Taguchi design, was performed using Minitab^®^ Statistical Software version 21.1 (Minitab LLC, State College, PA, USA; 2023). For the polyelectrolyte titration, a one-way analysis of variance (ANOVA) was performed on the ζ-potential data for all polysaccharide-to-liposome ratios separately for iota-carrageenan and fucoidan, followed by Tukey’s honestly significant difference (HSD) post hoc test for pairwise comparisons. Comparison of the in vitro release profiles of the different formulations was performed using the model-independent similarity factor (f2), with f2 < 50 considered indicative of a statistically significant difference between profiles. For in vitro cell uptake assays analyses StatView software (version 5.0) (SAS Institute, Cary, NC, USA) was used to apply analysis of variance (ANOVA). Statistically significant differences between the samples were determined using Fisher’s PLSD test. Lower than 0.05 *p*-values were considered statistically significant. Unless otherwise stated, all experiments were conducted as three independent biological replicates, and the results are presented as the mean ± standard deviation (SD).

## 3. Results and Discussion

### 3.1. Formulation of Xanthohumol-Loaded Liposomes

The first objective of this study was to optimize the formulation of xanthohumol-loaded liposomes in order to obtain nanocarriers with high drug entrapment efficiency, adequate colloidal stability, and sufficiently small particle size to allow subsequent surface functionalization. These characteristics are particularly important for the successful development of coated liposomal systems, as excessive vesicle size or poor stability may compromise both the coating process and the biological performance of the final formulation. To achieve this goal, we focused on key formulation variables expected to exert the greatest influence on the physicochemical properties of the liposomes, namely lipid concentration, lipid composition (DPPC:DOTAP ratio), and lipid-to-xanthohumol ratio. A 2^3^ + 1 full factorial experimental design was employed to investigate the individual and combined effects of these factors and to identify the optimal formulation conditions. Beyond selecting the most suitable liposomal formulation for subsequent surface modification, this approach also enabled a comprehensive evaluation of how each technological parameter affects vesicle size, ζ-potential, and xanthohumol entrapment efficiency, thereby providing insight into the formulation process. The matrix of the design, as well as the results of the factorial regression analysis, are presented in Table 1.

#### 3.1.1. Influence of the Varied Parameters on the Average Size

The data from the factorial regression analysis (Table 2) showed that the factor with the greatest influence on the average size of liposomes was the lipid concentration. A high positive effect value was observed (220.66), which indicated that an increase in concentration from 1 mM to 10 mM led to a statistically significant increase in the average size (*p*-value < 0.001). This can be explained by Smoluchowski’s theory of coagulation, which states that aggregation is proportional to the square of the particle concentration, a result also confirmed by other authors [29]. The Lipid phase:Xanthohumol ratio exerted a high positive effect (139.64) as well, indicating a significant increase in the average size when the ratio changed from 3:1 to 7:1 (a decrease in xanthohumol concentration). This was probably due to the positioning of xanthohumol at the boundary between the lipid tails and the polar head of the DPPC molecule, which in turn led to a reduction in the distance between the layers of the liposomes [30]. In addition, the presence of xanthohumol in liposome bilayers led to an increase in the fluidity of liposome membranes, decreasing the critical arrangement parameter (*p* < 1), which, from a thermodynamic point of view, contributed to the formation of smaller vesicles [31,32]. Conversely, at the lower xanthohumol content (7:1 ratio), fewer drug molecules are available to disrupt acyl-chain packing; the bilayer retains a more ordered, tightly packed, and rigid configuration characteristic of cholesterol-containing DPPC membranes, which favors lower membrane curvature and consequently the formation of larger vesicles. In addition, the reduced interfacial drug load at the 7:1 ratio diminishes the local disordering of the headgroup region, allowing a more extended interlamellar spacing and a larger overall hydrodynamic diameter. The net result is the observed increase in vesicle size with increasing Lipid phase:Xanthohumol ratio. The DPPC:DOTAP:Cholesterol ratio also had a statistically significant effect on the average size (*p* = 0.005), but the degree of influence was significantly lower compared to that of the other two individual factors. Regarding the combined effects of the variables on the average size, the data showed that one of the most significant interactions between the individual variables was that between lipid concentration and the lipid concentration:xanthohumol ratio. A significant positive synergistic effect (+110.06) was observed—a simultaneous increase in lipid concentration from 1 mM to 10 mM and a change in the Lipid phase:Xanthohumol ratio from 3:1 to 7:1 (decrease in xanthohumol concentration) led to a statistically significant increase in the average size of the liposomes (*p* < 0.001).

#### 3.1.2. Influence of the Varied Parameters on the ζ-Potential

As demonstrated by the data from the regression analysis (Table 3), the factor exerting the most substantial influence on the ζ-potential was the ratio between DPPC:DOTAP:CHL. The negative sign for the effect (−6.493) indicates that as the ratio transitions from 3:1:2 (high DOTAP concentration) to 7:1:2 (low DOTAP concentration), the value of the zeta potential undergoes a substantial decrease (*p* < 0.001). This outcome is predicated on the premise that DOTAP, being a cationic lipid, exerts a direct influence on ζ-potential [33]. Lipid phase:Xanthohumol ratio was another factor that significantly influenced the ζ-potential. The variable demonstrated a high effect on the ζ-potential (+6.16); however, it was observed that as the ratio increased from 3:1 to 7:1 (or as the concentration of xanthohumol decreased), there was a concomitant increase in the ζ-potential value. This trend suggests the presence of an interaction between xanthohumol and the polar group of DOTAP, resulting in a decrease in the overall value of ζ-potential. This phenomenon is likely attributable to the marginal deprotonation of xanthohumol, a process that results in the molecule acquiring a modest negative charge [34]. Although it showed a statistical significance (*p* = 0.004), the lipid concentration exerted a small effect on the ζ-potential (+1.47). The higher overall increase in the lipid concentration slightly increased the ζ-potential; however, compared to the other two parameters, the impact was negligible. The factorial regression analysis demonstrated that, from an interaction perspective, the most significant combined effect of the variables was that between DPPC:DOTAP:CHL and Lipid phase:Xanthohumol (*p* < 0.001), exhibiting a negative effect value (−2.23). This indicates that the degree to which Xanthohumol interacts with DOTAP is dependent on the concentration of DOTAP. The central point showed a statistical insignificance (*p* = 0.173), meaning that a linear relationship between the factors and the ζ-potential was observed.

#### 3.1.3. Influence of the Varied Parameters on the Entrapment Efficiency

The data from the factorial regression analysis (Table 4) indicated that the lipid concentration tends to be the dominant factor that affects the entrapment efficiency of xanthohumol (T = 22.93, *p* < 0.001). It is evident, by its high positive effect (+46.52) and high F-value (525.69), that as the lipid concentration was increased, a major increase in the entrapment efficiency was observed. This phenomenon can be attributed to the observation that, as the concentration of the phospholipids increases, bilayer volume and physical capacity increase, which drastically increases the incorporation of xanthohumol into the liposomes [10,35]. Another factor that had a statistically significant impact on the entrapment efficiency was the ratio between DPPC:DOTAP:CHL (T = 12.51, *p* < 0.001). The high positive effect (+25.39) demonstrates that as the ratio shifts from 3:1:2 to 7:1:2, the entrapment efficiency significantly increases. This was probably because DPPC formulations with higher concentrations were capable of forming a highly rigid ordered lipid matrix, which managed to sufficiently retain the hydrophobic Xanthohumol [31,36]. An interesting observation was that the Lipid phase:Xanthohumol ratio had no statistically significant effect on the entrapment efficiency as an individual parameter (*p* = 0.068); however, a strong interaction with the lipid concentration was observed (T = 6.98, *p* < 0.001), exhibiting a relatively high positive effect (+13.97). This clearly indicates that the concentration of xanthohumol, in the current levels of variation, does not influence the entrapment efficiency significantly, but when optimal concentration (7:1 ratio) was combined with high lipid concentration (10 mM), an overall high increase of the entrapment efficiency was observed. This was probably due to the bulky molecule of xanthohumol, which, at high concentration (3:1 ratio), in combination with low lipid concentration (1 mM), led to the destabilization of the liposomal membrane, which in turn could not retain the drug, leading to an overall lower entrapment efficiency [30]. Another interaction that significantly impacted the entrapment efficiency was the Lipid concentration x DPPC:DOTAP:CHL ratio (T = −3.68, *p* = 0.002). As previously discussed, the individual increase of both parameters enhances the entrapment efficiency. However, their simultaneous increase led to an overall decrease in the entrapment efficiency, as can be seen from the negative effect value (−7.47). This phenomenon may be attributed to the high rigidity of the DPPC molecule, which, when combined with high lipid concentration, limits the space in which the hydrophobic drug can be accommodated. The same effect was observed in structures with similar characteristics, like Xanthohumol [37].

#### 3.1.4. Optimization of the Results

All three investigated models describing vesicle size, entrapment efficiency, and ζ-potential demonstrated excellent statistical performance, with coefficients of determination (R^2^) ranging from 96.16% to 98.30% and adjusted R^2^ values remaining in close agreement with the corresponding R^2^ values (Table 5).

The high F-values obtained for all responses (56.27–129.91), together with *p*-values below 0.001, confirmed the overall significance of the models and indicated that the observed variations in liposome characteristics were primarily driven by the deliberately varied formulation parameters rather than by experimental noise. These results demonstrate that the developed models reliably describe the relationships between formulation variables and liposomal properties and can therefore be used for formulation optimization and selection of the most suitable candidate for subsequent surface modification.

**Figure 2:**
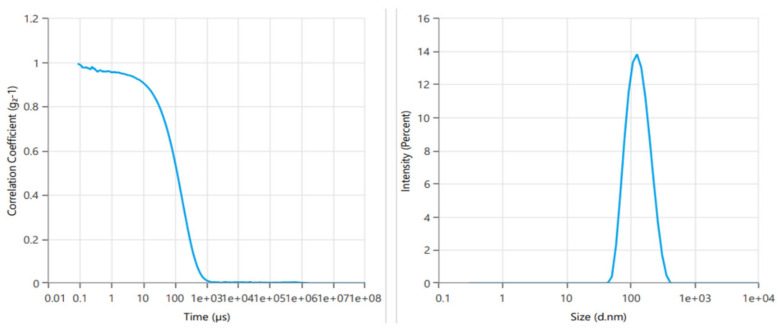
Size distribution based on intensity and correlogram of model UNC3.

The objective of the optimization process was to identify a liposomal formulation exhibiting a sufficiently small particle size for further surface functionalization, high ζ-potential to ensure colloidal stability and efficient electrostatic coating, and high xanthohumol entrapment efficiency. The focus of the experimental design was to formulate liposomes with an average size below 200 nm, suitable for further functionalization, high ζ-potential, and high entrapment efficiency of xanthohumol. All formulations showed ζ-potential values above 45 mV, indicating high colloidal stability and high charge density, appropriate for electrostatic surface modification. However, only three models exhibited average sizes below 200 nm: UNC1, UNC3, and UNC5 (Figure 2). Models UNC1 and UNC5 yielded insufficiently low drug entrapment efficiency (12.70 and 13.28, respectively). Therefore, the most suitable candidate for subsequent surface modification was determined to be model UNC3.

### 3.2. Surface Functionalization of the Xanthohumol-Loaded Liposomes

Following the optimization of the liposomal core formulation, the next objective of this study was to functionalize the liposome surface through coating with polysaccharides. All subsequent experiments were carried out on model UNC3, as it was selected as the optimal model from the previous experimental design. Two anionic biopolymers, iota-carrageenan (CAR) and fucoidan (FUC), were selected as coating materials with the aim of further enhancing the physicochemical stability of the vesicles while simultaneously imparting additional functionalities, including sustained drug release and potential “stealth-like” properties capable of reducing macrophage recognition and uptake. Such surface engineering is particularly attractive for xanthohumol-loaded liposomes, as prolonged circulation and protection from premature clearance may further improve the therapeutic potential of the encapsulated compound. To achieve this objective, the surface modification process was carried out in two consecutive stages. First, a polyelectrolyte titration approach was employed to determine the optimal polysaccharide-to-liposome ratio required for efficient surface coverage and electrostatic saturation of the vesicles. Establishing these ratios was essential for ensuring the formation of a complete and stable polymer shell around the liposomes. Subsequently, a Taguchi experimental design was applied to optimize the coating process by evaluating the influence of polysaccharide concentration, calcium ion concentration, and incubation time on the physicochemical characteristics of the resulting coated liposomes. This approach enabled both the identification of the optimal coating conditions and a detailed assessment of how the selected technological parameters affect vesicle size, ζ-potential, and colloidal homogeneity of the final formulations.

**Figure 3:**
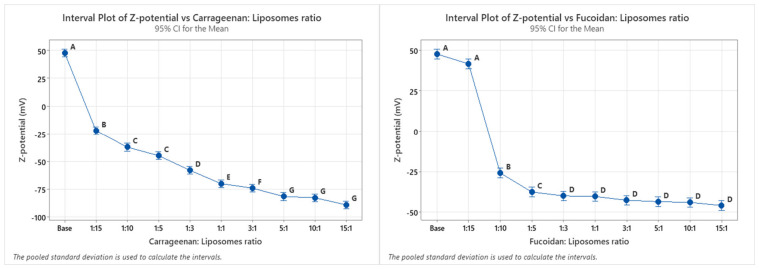
Interval plots of the ζ-potential, obtained from the polyelectrolyte titration. The letters represent statistical groups obtained by Tukey’s HSD post-hoc test *(Means that do not share a letter are significantly different)*.

In order to establish the optimal polysaccharide-to-liposomes ratio and the degree of surface saturation at which the liposomes become fully covered with polysaccharide, a polyelectrolyte titration method was employed, as previously outlined by Correa et al. (2019) with slight modifications [38]. Different Polysaccharide: Liposomes ratios were prepared, ranging from 15:1 to 1:15 (Figure 3). The ζ-potential values were statistically analyzed via One-way ANOVA, followed by Tukey’s HSD (Honest Significant Difference) post hoc test to perform multiple pairwise comparisons between the mean ζ-potential values of different ratios (Table 4). Differences were considered statistically significant at a probability value of *p* < 0.05. Data is presented as mean ± SD (*n* = 3).

As can be seen from Table 6, the ANOVA results showed that the Carrageenan: Liposomes ratio influenced the ζ-potential significantly (*p* < 0.001). It is evident that even at the first level of variation (1:15 Carrageenan: Liposomes), the ζ-potential shifts from distinctly positive (75.253) to highly negative (−22.227). The addition of iota-carrageenan led to a decrease in surface charge, thereby confirming the gradual adsorption of the anionic polysaccharide. Subsequent post hoc analysis yielded distinct statistical groupings (Groups A–G) corresponding to the varying degrees of surface coverage. The formulations with Carrageenan: Liposomes ratios of 5:1, 10:1, and 15:1 were all allocated to Group G, thereby indicating that their mean ζ-potential values (−81.8 mV to −89.3 mV) were not statistically different from one another (*p* > 0.05). This lack of significant difference confirmed that the electrostatic saturation of the liposomal surface was achieved at ratio of 5:1.

Regarding the fucoidan coating, one-way analysis of variance (ANOVA) showed a highly significant effect of the polysaccharide: liposomes ratio on the ζ-potential (*p* < 0.001). Tukey’s post hoc analysis identified distinct phases of the coating process (Figure 3). The initial incorporation of Fucoidan (1:15) led to a decrease in the ζ-potential from +75.25 mV to +51.55 mV, suggesting partial surface neutralization. A complete charge reversal was achieved at a Fucoidan: Liposomes ratio of 1:10, where the parameter shifted to −25.93 mV. Additional polysaccharide deposition resulted in a further decrease in surface charge until a saturation plateau was reached. The statistical grouping analysis revealed that Fucoidan: Liposomes ratios 1:3 through 15:1 all shared the same statistical letter (E), indicating that there was no statistically significant difference in their mean ζ-potential values (−40.09 mV to −46.02 mV). Consequently, 1:3 ratio was identified as the saturation point where the liposomal surface was effectively fully coated.

After evaluating the results from the polyelectrolyte titration, a Taguchi experimental design was applied in order to define the optimal settings for the preparation of surface-modified liposomes, stabilized by cross-linking the coating, and to determine how the polysaccharide concentration, the concentration of CaCl_2,_ and the time of incubation influence the average size, ζ-potential, and polydispersity index. Table 7 represents the design matrix and the measured corresponding characteristics of the modified liposomes, and Table 6 presents the results from the analysis of variance.

#### 3.2.1. Effect of the Varied Parameters on the Average Size of the Coated Liposomes

The data from the Taguchi L9 design and the subsequent regression analysis (Table 8) revealed distinct and contrasting patterns in the effect of the variables on the average vesicle size. The Taguchi signal-to-noise ratio analysis for iota-carrageenan revealed that both the polysaccharide and CaCl_2_ concentrations showed equally strong positive effects on the average size, whereas incubation time influenced the average size to a lesser degree. In contrast, the analysis of the fucoidan system showed dominance of the CaCl_2_ concentration, compared to the much lower contribution of the polysaccharide concentration and incubation time.

The high impact of the iota-carrageenan concentration can be explained by the physicochemical behaviour of the polymer at the liposomal surface. Iota-carrageenan is a linear, double-helix-forming polysaccharide that adsorbs onto the liposome surface and forms a hydrated, gel-like coating layer [39,40]. As the polymer concentration increases from 0.5 to 2.0 mg/mL, the adsorbed layer becomes progressively thicker. This directly increases the hydrodynamic diameter, as measured by dynamic light scattering (DLS). At higher concentrations, free polymer chains in the bulk solution may additionally engage in bridging flocculation between adjacent liposomes, temporarily or permanently linking particles, which results in a further increase in the average size [41]. The influence of the CaCl_2_ concentration can be explained by the cation-specific gelation of the iota-carrageenan, which, unlike other carrageenans, undergoes an ordered crosslinking specifically in the presence of Ca^2+^ ions, coordinating between opposing sulfate groups on adjacent helical chains [42]. As CaCl_2_ concentration increases from 1 to 10 mM, this crosslinking intensifies both within the surface coating and between the coatings of adjacent liposomes, expanding the effective hydrodynamic layer and promoting inter-particle bridging.

The sensitivity of the average size of the fucoidan-coated liposomes to CaCl_2_ concentration can be explained by the molecular architecture of fucoidan. As a highly sulfated, branched fucose-rich polysaccharide with one of the highest anionic charge densities among marine polysaccharides, fucoidan interacts with Ca^2+^ ions via a non-specific, electrostatically driven mechanism rather than the geometrically ordered, helix-stabilizing coordination characteristic of iota-carrageenan [43,44]. The dense distribution of sulfate groups across the fucoidan backbone provides numerous binding sites for divalent calcium, and even a minor increase in Ca^2+^ concentration generates dense, disordered crosslinks between fucoidan chains adsorbed on adjacent liposome surfaces. This results in a rapid, concentration-dependent inter-particle bridging and aggregation process that is far more pronounced and less controllable than the structured gel network formed by iota-carrageenan [45].

#### 3.2.2. Effect of the Varied Parameters on the Polydispersity Index (PDI) of the Coated Vesicles

The data from the signal-to-noise ratio shows that for iota-carrageenan model, all three factors show relatively similar levels of ∆-values, which signifies the balanced contribution of all three factors to the levels of PDI. The regression analysis confirms these results, with high F-values for all three factors and *p* < 0.001. A clear trend can be seen for an increase in the PDI value with the increase of polysaccharide and CaCl_2_ concentrations. When iota-carrageenan is added at low concentrations (0.5 mg/mL), the amount of polymer available per liposome is limited, and adsorption is governed by the most energetically favourable surface interactions. Under these conditions, the coating process tends to be relatively self-equalising: polymer preferentially adsorbs onto the most receptive surface sites, and the resulting coating layer is thin and comparatively uniform across particles. As the polysaccharide concentration increases to 1.0 and then 2.0 mg/mL, the excess iota-carrageenan in the solution increases the probability of multilayer deposition and polymer chain entanglement at the liposome surface [46]. This is probably due to the fact that at higher concentrations, the local polymer concentration around individual liposomes becomes more heterogeneous. Liposomes surrounded by a higher local density of polymer chains at the moment of mixing acquire thicker initial coatings than the microenvironments with lower polysaccharide concentrations [47].

The mechanism through which CaCl_2_ affects PDI operates through multiple parallel pathways, all related to the specificity and extent of iota-carrageenan crosslinking. Iota-carrageenan is unique among carrageenan subtypes in its ability to undergo ordered, cation-specific gelation in the presence of Ca^2+^. The crosslinking mechanism involves Ca^2+^ ions coordinating between the sulfate groups on opposing double-helical iota-carrageenan chains, thereby stabilising a three-dimensional gel network. At a concentration of 1 mM CaCl_2_, the crosslinking reaction is limited in extent, sufficient only to stabilize the adsorbed polymer layer against desorption, but insufficient to create a dense, rigid gel network. The crosslinks that form are relatively sparse and distributed across the liposome surface in a pattern that is broadly similar from particle to particle. This is because the low Ca^2+^ activity means that only the highest-affinity sulfate coordination sites are occupied. The resulting coating variability across the liposome population is limited, and PDI remains low. As the CaCl_2_ concentration increases to 5 mM and then 10 mM, the crosslinking density increases rapidly. The resulting liposome population is heterogeneous in both coating thickness and hydrodynamic diameter, thereby increasing PDI [48].

An opposing trend was observed for the incubation time. As the incubation time increased from 30 min to 120 min, a general decrease in the value of the PDI was observed. The explanation necessitates the differentiation between two kinetically separable stages of polysaccharide adsorption: the initial adsorption phase and the subsequent equilibration phase. During the initial adsorption phase, which occurs predominantly within the first 30 min of incubation, iota-carrageenan chains rapidly associate with the liposome surface. This is driven by electrostatic attraction between the anionic sulfate groups of the polysaccharide and the cationic lipid headgroups, as well as hydrophobic interactions and van der Waals forces. This initial adsorption is rapid but random. Chains settle on the liposome surface in a kinetically determined configuration that reflects local polymer concentration gradients, the orientation of the chain at the moment of surface contact, and the availability of surface binding sites. The resultant coating layer is found to be heterogeneous. During the subsequent equilibration phase, the adsorbed polymer layer undergoes progressive thermodynamic relaxation. Chains that adsorb in extended, partially hanging configurations rearrange into more compact trains-and-loops structures, thus maximising contact with the surface and minimising conformational entropy penalties. Chains that initially adsorbed on overcrowded surface regions undergo partial desorption and re-adsorption onto under-covered regions of the same or adjacent liposomes. By 120 min, the system has advanced sufficiently along this equilibration pathway that the reduction in PDI is both substantial and statistically highly significant [49].

For the fucoidan model, the incubation time was the most dominant factor, while the CaCl_2_ concentration also affected the PDI significantly. Unlike the iota-carrageenan PDI, which followed a predominantly linear, well-balanced response surface amenable to straightforward regression modelling, the fucoidan PDI was characterised by non-monotonic factor responses and a reversal of factor hierarchy relative to fucoidan particle size. The incubation time main effect plot shows non-linear effect regarding the PDI. It can be seen that the PDI decreased when the incubation time was increased from 30 to 60 min, and practically remained the same when moving to 120 min of incubation time. The sharp improvement within the first 60 min reflects fucoidan’s rapid adsorption kinetics. Unlike iota-carrageenan, which forms a structured helical gel network that continues to reorganise over timescales exceeding 2 h and equilibrates slowly, fucoidan adsorption is driven by strong non-specific electrostatic interactions that quickly and efficiently bring polymer chains to the liposome surface [50]. Within 30 min, most of the adsorption has occurred, but the distribution of the coating across the liposome population is still non-uniform, reflecting the stochastic nature of the initial polymer–surface contacts. By 60 min, two processes have probably operated sufficiently to produce a measurably more uniform coating. Firstly, the primary adsorption layer has had time to equilibrate spatially—chains that initially adsorbed in non-optimal, kinetically trapped configurations have partially rearranged to maximise surface contact and reduce intra-particle heterogeneity in the coating. Secondly, and more importantly for PDI, liposomes that acquired less polymer during the initial adsorption burst have had time to recruit further polymer from the bulk, thereby reducing variation in total coating mass between particles.

A similar trend was observed for the effect of CaCl_2_ concentration on the PDI. The PDI shows a tendency to decrease when moving from 1 mM to 5 mM. However, when the CaCl_2_ concentration was increased to 10 mM, the PDI value increased. This can be explained by the fact that, at 1 mM, the fucoidan coating is insufficiently cross-linked to achieve mechanical stability. The sulfate-Ca^2+^-sulfate coordination bonds that constitute the crosslinks are sparse, and the polymer chains adsorbed onto the liposome surface retain substantial conformational mobility. Under these conditions, the coating is dynamic rather than fixed—the chains continue to adsorb, desorb and rearrange throughout the incubation period, leading to an insufficient crosslinking density. This can also mean that the outer regions of the adsorbed polymer layer are not rigidly anchored and extend variably into solution, contributing to variation in hydrodynamic diameter between particles. At 5 mM CaCl_2_, the crosslinking density achieves an optimal balance. There is sufficient Ca^2+^ to crosslink the fucoidan layer on every liposome in the suspension rapidly and simultaneously, creating a coherent, mechanically stable coating with relatively little particle-to-particle variation. The crosslinking is dense enough to rigidify the outer polymer layer and reduce its variability in hydrodynamic extension, but not so dense as to drive significant rates of inter-particle bridging reactions. The result is the most uniform coating achievable across the liposome population within the tested parameter space. At 10 mM CaCl_2_, the excess Ca^2+^ drives non-specific, electrostatically driven bridging aggregation, which was identified as the dominant mechanism governing fucoidan particle size at high Ca^2+^ concentrations. Fucoidan’s dense sulfate groups provide numerous non-specific Ca^2+^ binding sites and, at 10 mM, the Ca^2+^ activity is sufficient to simultaneously bridge sulfate groups on polymer chains adsorbed on adjacent liposome surfaces. These inter-particle crosslinks create permanent or semi-permanent dimers, trimers, and small clusters that register as large-diameter particles in dynamic light scattering (DLS). The resulting population is extremely heterogeneous, spanning a wide range from individual coated liposomes to multi-particle aggregates, which produces the sharp PDI deterioration observed at 10 mM [51,52,53].

#### 3.2.3. Effect of the Varied Parameters on the ζ-Potential of the Modified Liposomes

The data for the ζ-potential shows that both models showed solely negative values of the ζ-potential, which can mean that the cross-linking did not affect the stability of the coatings. The signal-to-noise ratio analysis showed that for both models, the CaCl_2_ concentration was the most dominant factor that affected the value of the ζ-potential, showing ∆-value of 10.56 for the iota-carrageenan model and ∆-value of 0.85 for the fucoidan model. In both models, the regression analysis showed high F-values for the parameters, while *p*-value < 0.001 was present for both of the models.

The importance of Ca^2+^ ions for the ζ-potential was expected and can be explained by the anionic nature of the polysaccharides. In both cases, as the concentration of CaCl_2_ was increased, an overall reduction in the ζ-potential was observed. The difference between the two polysaccharides is that in the fucoidan model, when the concentration of CaCl_2_ was increased, a slight progressive decrease of the ζ-potential was observed, as can be seen from the signal-to-noise ratio analysis. In contrast, for the iota-carrageenan model, a sharp decrease in the value of the ζ-potential was observed when the concentration shifted from 1 mM to 5 mM. This might be due to the nature of Ca^2+^- polysaccharide interaction. For iota-carrageenan, Ca^2+^ ions participate in a geometrically specific, cooperative coordination reaction with the sulfate groups of double-helical chain segments. The helical secondary structure of iota-carrageenan preorganizes pairs of sulfate groups into optimal positions for Ca^2+^ coordination, and the binding of each Ca^2+^ ion stabilizes adjacent helical segments and promotes further Ca^2+^ binding in a cooperative cascade. Once the Ca^2+^ concentration exceeds a threshold, the extent of sulfate coordination—and therefore charge neutralization—increases rapidly and non-linearly, producing the sharp ζ-potential collapse observed between 1 and 5 mM [42].

For fucoidan, no such cooperative mechanism exists. Fucoidan does not form regular secondary structures in a solution, and its sulfate groups are distributed in a disordered, three-dimensional arrangement across its branched architecture rather than being pre-organized by a helical template. Ca^2+^ ions interact with fucoidan sulfate groups through non-specific electrostatic coordination—each Ca^2+^ binds independently to whichever sulfate groups happen to be in its immediate vicinity, without the propagating, helix-stabilizing mechanism that amplifies the effect in iota-carrageenan. The binding affinity per site is lower, the binding is less geometrically specific, and the events are statistically independent rather than cooperative [45,54] The consequence is a linear, gradual, and quantitatively minor neutralization of surface charge.

For the fucoidan model, apart from the CaCl_2_ concentration, the polymer concentration also exerted statistically significant effect on the ζ-potential. As can be seen from the signal-to-noise analysis ratio, as the concentration was increased from 0.5 to 2.0 mg/mL, a relatively small increase in S/N value was observed, meaning the ζ-potential increased. The regression analysis showed a relatively high F-value of 16.87 and *p*-value < 0.001. The effect can be explained by the fact that more fucoidan on the liposome surface means more sulfate groups exposed at the electrokinetic shear plane, which shifts the zeta potential to more negative values.

#### 3.2.4. Optimization of the Results from the Liposomes Coating

From the Taguchi analysis, it is evident that for both formulations, the concentration of CaCl_2_ was one of the most influential factors. The concentration of CaCl_2_ increased the average size and PDI, while also decreasing the ζ-potential; thus, for both models, the level of the variable was set to its lowest (1 mM). For the CAR model, the average size as well as the PDI increased significantly as the polysaccharide concentration increased. The ζ-potential was found to increase with the increase of the polysaccharide concentration; however, the effect was determined to be statistically insignificant. Therefore, the optimal level of polysaccharide concentration for the CAR model was set to level 1 (0.5 mg/mL). For the FUC model, the polysaccharide concentration had a very highly significant impact on the ζ-potential, as the increase in the concentration led to an increase in the value of the parameter. While fucoidan concentration had no significant impact on the PDI, it showed the opposite effect on the average size, as the average size increased with the increase in the polymer concentration. Thus, level 2 (1.0 mg/mL) was selected as the most appropriate level. The incubation time showed almost no significant effect on most of the parameters, with the exception of the PDI. In CAR model, the longer the incubation time was set, the lower the value of PDI was observed, so level 3 (120 min) was set as the optimal level. For the FUC model, the lowest PDI was observed at level 2 (60 min), so this level was selected as optimal.

**Figure 4:**
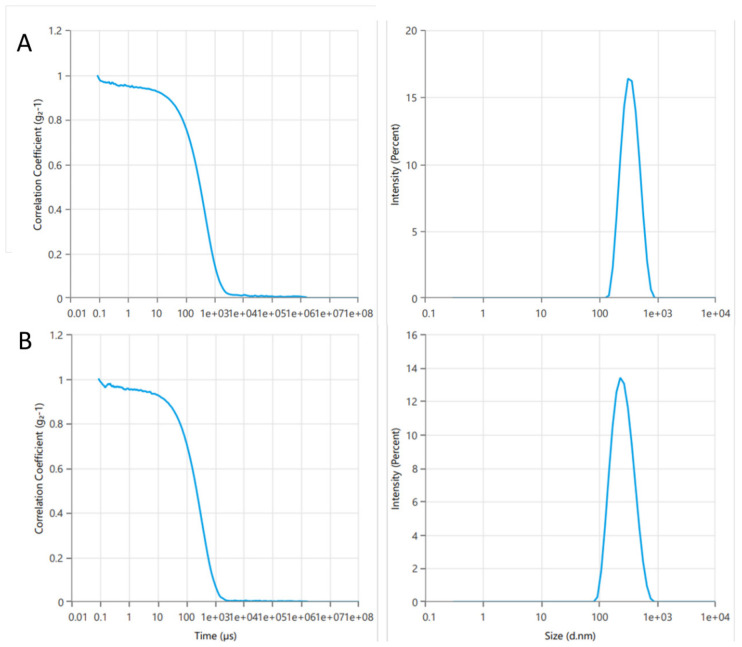
Size distribution based on intensity and correlogram of the formulated optimal models of iota-carrageenan (**A**) and fucoidan (**B**) modified liposomes.

As a result of the performed coating optimisation, new liposome models were prepared and characterized by the aforementioned techniques, and the drug entrapment efficiency was also determined to evaluate whether the optimal model kept the initial amount of XN. The following results were observed: CAR optimal model (0.5 mg/mL polysaccharide concentration, 1 mM CaCl_2_ concentration, and 120 min incubation time) showed an average size of 331.4 ± 2.1 nm, PDI 0.221 ± 0.008, ζ-potential value of −33.09 ± 2.69 mV. FUC model (1.0 mg/mL, 1 mM CaCl_2,_ and 60 min incubation time) yielded 233.3 ± 3.39 nm, PDI 0.160 ± 0.004, ζ-potential −24.95 ± 1.85 mV. The high drug entrapment efficiency, 98.57% ± 0.25 for FUC model and 99.78 ± 0.25 for CAR model, shows that after encapsulation, practically the whole amount of the drug was retained within the liposomes. The vesicle size distribution curves of the formulated optimal models of iota-carrageenan and fucoidan modified liposomes are presented in Figure 4.

### 3.3. In Vitro Release of Xanthohumol from the Formulated Liposomes

**Figure 5:**
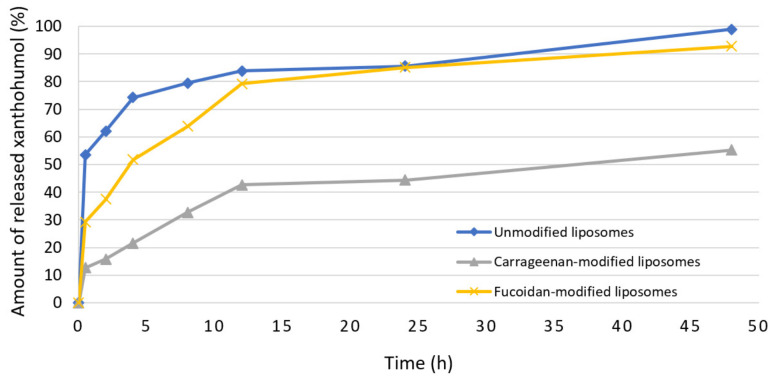
Cumulative plot of XN drug release from unmodified, iota-carrageenan-modified, and fucoidan-modified liposomes.

The in vitro release profiles of xanthohumol from unmodified liposomes (UNC), carrageenan-modified (CAR), and fucoidan-modified (FUC) liposomes cross-linked with calcium ions were evaluated over 48 h. The cumulative release data is presented in Figure 5. The release of xanthohumol from the unmodified liposomes exhibited a burst release as approximately 55% of the incorporated xanthohumol was released within 1 h and around 75% after 4 h, followed by a plateau release reaching 98% by 48 h. The release from fucoidan-modified liposomes followed a slower release of xanthohumol at the early hours (around 28% released within 1 h and around 65% released after 6 h); however, at the later stages of the assay, the drug release became faster, and by the 12th h, it reached around 80% and approximately 90% at the 48th h. This shows that the fucoidan coating effectively reduces the burst effect, potentially preventing dose dumping, while still allowing a near-complete release over 48 h.

The iota-carrageenan coating provided slower drug release (around 10% at the 1st h, around 35% at the 6th h, and around 55% at the 48th h). This shows that the cross-linked iota-carrageenan coating creates a strong barrier that significantly slows the drug release, providing a good opportunity for a system for prolonged or targeted drug delivery. The significantly slower drug release from the iota-carrageenan can be attributed to the specific helical structure of the polysaccharide, which, when cross-linked with Ca^2,^, produces a tight, cohesive hydrogel shell [42], while the fucoidan chain has a more heterogeneous and branched structure, which, when cross-linked with Ca^2+^, produces a looser and less organized network [55,56] It should be noted that the saturation solubility of xanthohumol in the release medium was not experimentally determined; therefore, strict sink conditions could not be formally verified throughout the experiment. Nevertheless, all formulations were evaluated under identical experimental conditions specifically designed to promote sink-like behaviour, allowing a reliable comparative assessment of their release performance. Consequently, the obtained release profiles should be interpreted as comparative rather than absolute release rates and should not be considered as an in vitro–in vivo correlation.

The data for the in vitro drug release was fitted to mathematical models, which are used to describe drug release kinetics process. The R^2^–values of the corresponding models are presented in Table 9.

The data from the mathematical modelling demonstrated that the release of xanthohumol from the unmodified liposomes can be described by first-order kinetic model (R-sq = 0.910), which supports the rapid, concentration-driven release of the substance from the vesicular structure. The Korsmeyer-Peppas model demonstrated the optimal fit for both modifications (iota-carrageenan and fucoidan), with R^2^-values of 0.964 and 0.994, respectively. For both models, the release exponent was determined to be lower than 0.43, and slightly higher for the fucoidan model (n = 0.414) compared to the iota-carrageenan one (n = 0.405), which shows that for both models the primary mechanism of drug release can be considered to be Fickian diffusion with n-values typical for spherical systems [57]. Additionally, low R-sq values were observed for the zero-order model, thereby confirming that the rate of xanthohumol release is not constant and time-independent. Also, the Hixson-Crowell model performed poorly with R-sq < 0.80 across all three formulations, which also confirms the absence of substantial erosion of the carriers under the employed dissolution conditions.

To compare the release profiles of the three formulations, the model-independent similarity factor (f2) was calculated for each pair, following the convention of including all time points up to the first at which either profile exceeded 85% release. All three pairwise comparisons yielded f2 values well below 50—UNC vs. CAR, f2 = 17.3; UNC vs. FUC, f2 = 37.0; and CAR vs. FUC, f2 = 25.7—confirming that the three release profiles are different from one another. These results support the observation that both coatings significantly modified xanthohumol release relative to the uncoated liposomes, with the iota-carrageenan coating producing the most pronounced retardation and the two coatings differing significantly from each other.

### 3.4. In Vitro Macrophage Uptake Assay for the Formulated Liposomes

Following the physicochemical characterization of the coated liposomal formulations, their biological activity was evaluated by assessing cellular uptake in macrophages. One of the major challenges associated with conventional liposomal carriers is their rapid recognition and clearance by cells of the mononuclear phagocyte system, which can significantly reduce circulation time and limit therapeutic efficacy. Since one of the primary objectives of the present study was to develop polysaccharide-coated liposomes with potential “stealth-like” properties, it was essential to investigate whether the introduced surface modifications could reduce macrophage recognition and internalization.

#### 3.4.1. Effect of Polysaccharide Coatings on RAW264.7 Macrophage Uptake

**Figure 6:**
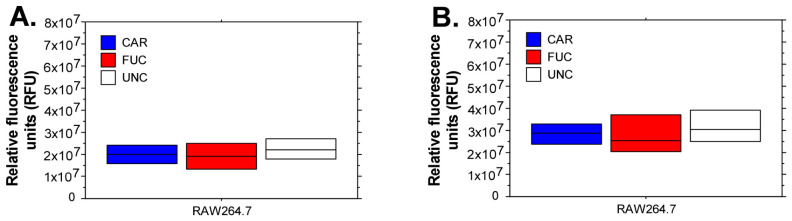
In vitro macrophage uptake of unmodified liposomes (UNC), iota-carrageenan modified (CAR), and fucoidan modified (FUC) at concentration of 10 µg/mL for a period of 1 h (**A**) and 2 h (**B**). The center line in the boxes indicates the median of the data.

To evaluate the effect of iota-carrageenan (CAR) and fucoidan (FUC) surface coatings on liposome internalisation by macrophages, RAW264.7 cells were incubated with coated and uncoated (UNC) liposomes at concentrations of 10 and 50 µg/mL for 1 and 2 h, and uptake was quantified by fluorometry as relative fluorescence units (RFU). At the lower concentration of 10 µg/mL, no statistically significant differences in cellular uptake were observed between CAR, FUC, and UNC liposomes at either the 1-h or 2-h time point (*p* > 0.05 for all comparisons) (Figure 6). RFU values across all three groups ranged from approximately 1.3 to 3.9 × 10^7^, with broadly overlapping distributions at both time points. A modest increase in uptake was observed from 1 h to 2 h across all groups, consistent with ongoing active endocytosis (Figure 6B).

**Figure 7:**
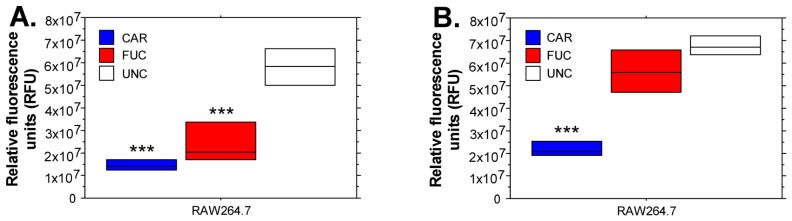
In vitro macrophage uptake of unmodified liposomes (UNC), iota-carrageenan modified (CAR), and fucoidan modified (FUC) at concentration of 50 µg/mL for a period of 1 h (**A**) and 2 h (**B**) *** *p* < 0.001. The center line in the boxes indicates the median of the data.

At 50 µg/mL, distinct and statistically significant differences in cellular uptake emerged between coated and uncoated liposomes (Figure 7). At the 1-h time point, both CAR- and FUC-coated liposomes demonstrated markedly reduced uptake by RAW264.7 cells, compared to UNC liposomes, with mean RFU values of approximately 1.5 × 10^7^ and 2.5 × 10^7^, respectively, versus 5.8 × 10^7^ for UNC (*p* < 0.001 for both comparisons). This represents an approximate 74% reduction in uptake for CAR-coated liposomes and a 57% reduction for FUC-coated liposomes relative to uncoated controls at 1 h (Figure 7A).

At the 2-h time point, the two coatings diverged markedly in their behaviour. CAR-coated liposomes maintained highly significant reduction in uptake compared to UNC (*p* < 0.001), with a mean RFU of 2.2 × 10^7^ versus 6.8 × 10^7^ for UNC. In contrast, FUC-coated liposomes exhibited a dramatic increase in uptake between 1 and 2 h, rising from mean RFU of 2.5 × 10^7^ to 5.7 × 10^7^ RFU, becoming statistically indistinguishable from UNC liposomes at this time point (*p* > 0.05). These findings indicate that iota-carrageenan confers stable, time-independent phagocytic evasion in macrophages, whereas fucoidan provides only transient early evasion that is subsequently reversed.

**Figure 8:**
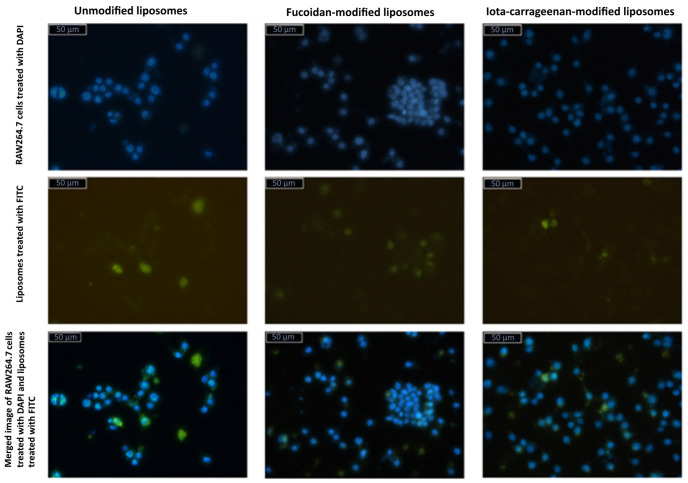
Fluorescent microscopy of macrophage uptake of unmodified liposomes (UNC), iota-carrageenan modified (CAR), and fucoidan modified (FUC) at concentration of 50 µg/mL.

To complement the quantitative data obtained by fluorometric measurements and confirm genuine intracellular uptake, RAW264.7 cells incubated with CAR, FUC, and UNC liposomes at 50 µg/mL for 2 h were examined by fluorescence microscopy using DAPI nuclear staining and FITC-labelled liposomes. Merged DAPI/FITC overlay images revealed a clear and visually consistent gradient of FITC signal intensity across the three formulations: CAR < FUC < UNC (Figure 8). In cells incubated with UNC liposomes, bright FITC signal was observed in approximately 50–60% of DAPI-positive cells, distributed densely across the entire field of view. FUC-coated liposomes produced moderate FITC signal in approximately 20–25% of cells, while CAR-coated liposomes exhibited sparse signal in only approximately 10% of cells.

**Figure 9:**
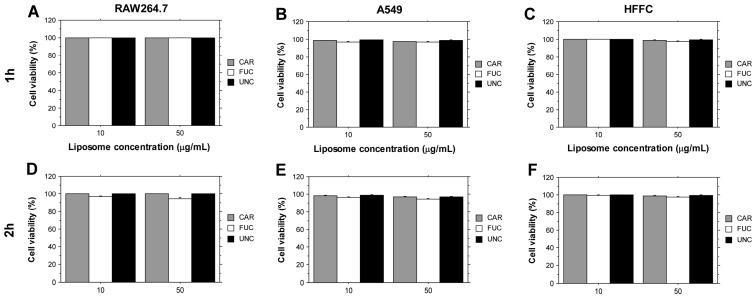
Cell viability of RAW264.7 macrophages, A549 lung adenocarcinoma cells, and HFFC fibroblasts following treatment with uncoated (UNC), iota-carrageenan-coated (CAR), and fucoidan-coated (FUC) liposomes at 10 and 50 µg/mL for 1 h (**A**–**C**) and 2 h (**D**–**F**). Viability is expressed as a percentage relative to untreated control cells. Data are presented as mean ± SD (n = 3).

To exclude the possibility that the differences in fluorescence signal observed in the uptake assay reflected differential cytotoxicity rather than genuine differences in internalisation, cell viability was assessed for all three formulations at both concentrations and time points. As shown in Figure 9, none of the formulations significantly affected the viability of RAW264.7, A549, or HFFC cells: viability remained at approximately 95–100% across all conditions, concentrations (10 and 50 µg/mL), and incubation times (1 and 2 h). Importantly, viability did not differ appreciably between uncoated and coated liposomes, confirming that the markedly higher uptake of uncoated liposomes by RAW264.7 macrophages cannot be attributed to reduced cytotoxicity of the coated formulations. These results confirm that the differences in fluorescence signal reflect genuine differences in liposome internalisation rather than differential effects on cell survival.

In all three conditions, the FITC signal appeared as discrete punctate spots rather than diffuse membrane fluorescence, a pattern characteristic of vesicular endosomal compartmentalisation following active endocytosis [58,59]. The absence of nuclear colocalization with the DAPI channel confirmed that FITC signal was confined to the cytoplasm, consistent with liposome sequestration within endosomes or lysosomes. These microscopy findings independently corroborate the results from fluorometric measurements and support the conclusion that the quantified RFU differences reflect genuine differences in intracellular liposome accumulation rather than surface adhesion artefacts.

The gradient of CAR evasion across the tested cell line suggests that the absolute magnitude of evasion scales with the active phagocytic capacity of the target cell: in cells with high phagocytic activity, the steric barrier effect produces large RFU differences relative to uncoated controls, whereas in cells with low baseline uptake, the same physicochemical effect is present, but differences are too small to reach statistical significance. In contrast, the fucoidan coating generates sufficient surface hydrophilicity, at early time points, to transiently reduce non-specific uptake. The subsequent loss of this protective effect over time may arise from scavenger receptor clustering and signalling amplification, leading to active recognition of the fucoidan-coated surface as a pathogen-associated molecular pattern (PAMP), ultimately driving receptor-mediated endocytosis that exceeds the protective steric effect; however, this mechanism is hypothetical and would require dedicated mechanistic experiments that can confirm [60].

#### 3.4.2. Uptake of Surface-Modified Liposomes by Non-Phagocytic HFFC Fibroblasts and A549 Lung Epithelial Cells

**Figure 10:**
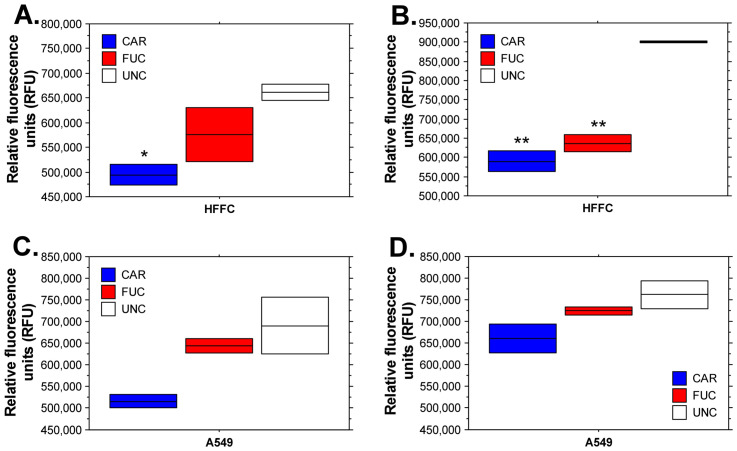
In vitro uptake of unmodified liposomes (UNC), iota-carrageenan modified (CAR), and fucoidan modified (FUC) at concentration of 50 µg/mL for a period of 1 h (**A**,**C**) and 2 h (**B**,**D**), by non-phagocytic cells (A549 and HFFC). * *p* < 0.05, ** *p* < 0.01. The center line in the boxes indicates the median of the data.

To assess whether the observed uptake effects were specific to professional phagocytes or reflected broader physicochemical properties of the coatings, HFFC human foreskin fibroblast cells were incubated with FITC-labeled liposomes under identical conditions (Figure 10). RFU values in HFFC cells were consistently approximately 100-fold lower than those recorded in RAW264.7 macrophages across all conditions, reflecting the non-phagocytic nature of fibroblasts and confirming the validity of the experimental system.

At 50 µg/mL and 1-h incubation (Figure 10A), CAR-coated liposomes demonstrated significantly reduced uptake compared to UNC (*p* < 0.05), with a mean RFU of approximately 495,000 versus 662,000 for UNC. FUC-coated liposomes did not differ significantly from UNC at this time point (*p* > 0.05). At 2 h, both coatings reached statistical significance versus UNC (*p* < 0.01 for both CAR and FUC), with mean RFU values of approximately 590,000 and 637,000, respectively, compared to approximately 900,000 for UNC (Figure 10B).

A549 human lung adenocarcinoma cells were included as a second non-phagocytic reference cell line with relevance to tumor drug delivery. RFU values were in the same order of magnitude as those recorded for HFFC, ranging from approximately 500,000 to 760,000 across all groups and time points, consistent with low-level non-specific endocytosis. Neither CAR nor FUC coating reached statistical significance versus UNC at either the 1-h or 2-h time point (*p* > 0.05 for all comparisons), indicating that polysaccharide coating effects are not detectable at a statistically significant level in this cell line under the conditions tested (Figure 10C,D). A progressive increase in CAR-coated liposome uptake was observed from 1 h (~515,000) to 2 h (~660,000), representing a 28% increase, suggesting that the coating delays but does not prevent slow non-specific endocytosis in lung adenocarcinoma cells.

The absence of statistically significant differences between coated and uncoated liposomes at 10 µg/mL, in contrast to the highly significant effects observed at 50 µg/mL, indicates that a minimum concentration threshold is required to elicit measurable coating-dependent modulation of macrophage uptake. This concentration dependence is consistent with previous reports demonstrating that surface coating effects on liposomal uptake are ligand density-dependent, requiring sufficient coating mass to generate meaningful steric or receptor-engagement effects [61,62]. At sub-threshold concentrations, the number of surface-bound polysaccharide molecules may be insufficient to either sterically shield the liposome surface from opsonisation or to present adequate ligand density for pattern recognition receptor engagement.

## 4. Conclusions

In the present work, xanthohumol-loaded cationic liposomes were successfully developed, surface-modified with the marine polysaccharides, iota-carrageenan and fucoidan, and stabilized by ionotropic cross-linking with Ca^2+^ ions. The 2^3^ + 1 full factorial design demonstrated that the lipid concentration and the DPPC: DOTAP: cholesterol ratio were the dominant determinants of vesicle size, ζ-potential, and xanthohumol entrapment efficiency, with all three response models displaying high statistical reliability (R^2^ > 89%). Polyelectrolyte titration identified saturation of the liposomal surface at a 5:1 carrageenan-to-liposome ratio and at a 1:3 fucoidan-to-liposome ratio, while subsequent Taguchi L9 optimization revealed clearly distinct behaviours of the two polysaccharides during cross-linking: the iota-carrageenan shell was governed by a balanced contribution of polysaccharide concentration, CaCl_2_ concentration, and incubation time, consistent with cooperative, helix-templated Ca^2+^ coordination, whereas fucoidan was overwhelmingly dominated by CaCl_2_ concentration, in agreement with non-specific electrostatic crosslinking of its branched, sulfate-rich backbone.

The two cross-linked coatings translated these structural differences into pronounced differences in functional performance. Compared with the burst-type release of uncoated liposomes (≈55% within the 1st hour), the fucoidan coating reduced the early release of xanthohumol while still allowing near-complete release over 48 h, whereas the iota-carrageenan coating produced a markedly slower, sustained release (≈55% at 48 h) that was best described by Korsmeyer-Peppas, indicating a robust hydrogel-like diffusion barrier. In vitro uptake studies in RAW264.7 macrophages further demonstrated that, at a concentration of 50 µg/mL, both coatings substantially suppressed phagocytic internalization at 1 h, but only the iota-carrageenan shell sustained this protective effect at 2 h, while fucoidan-coated vesicles reverted to uptake levels indistinguishable from uncoated controls—possibly reflecting scavenger-receptor-mediated recognition of the highly sulfated fucoidan surface. The reduced uptake observed in non-phagocytic HFFC fibroblasts and the absence of significant differences in A549 epithelial cells confirmed that the protective effect of the coatings scales with the active phagocytic capacity of the target cell rather than reflecting a non-specific physicochemical barrier.

Overall, these results show that cross-linked iota-carrageenan coating presents an effective surface-engineering strategy for xanthohumol-loaded liposomes, simultaneously providing controlled and prolonged drug release and stable, time-independent evasion of macrophage uptake—features consistent with genuine “stealth-like” behaviour. Fucoidan coating, although less robust as long-term phagocytic shield, may still be of interest for applications in which a moderated burst effect combined with eventual macrophage targeting is desirable. It should be acknowledged that the hydrodynamic diameter of the optimal iota-carrageenan model (≈331 nm) lies above the size range most commonly associated with efficient passive, EPR-mediated tumour accumulation; however, passive extravasation is only one of several routes by which nanocarriers may reach tumour tissue, and the macrophage-evasion and controlled-release properties demonstrated here are expected to benefit systemic delivery independently of any single targeting mechanism. The platform described here therefore offers a versatile and tuneable basis for the further development of polysaccharide-coated liposomal carriers for the systemic or targeted delivery of fragile lipophilic actives such as xanthohumol and warrants further evaluation in pharmacokinetic and efficacy studies in vivo.

## Figures and Tables

**Table 1:** Design matrix with the formulation variables and corresponding xanthohumol-loaded liposomes model (*n* = 3).

Model	Lipid Concentration		DPPC:DOTAP:CHL		Lipid Phase:XN		Average Size ± SD (nm)	Ζ-Potential ± SD (mV)	Entrapment Efficiency (%)
	Level	mM	Level	Ratio	Level	Ratio			
UNC1	−1	1	−1	3:1:2	−1	3:1	178.9 ± 2.4	51.4 ± 2.1	12.7 ± 0.1
UNC2	+1	10	−1	3:1:2	−1	3:1	271 ± 26	53.4 ± 0.7	62.4 ± 1.8
UNC3	−1	1	+1	7:1:2	−1	3:1	163 ± 2	47.6 ± 0.8	56.7 ± 0.3
UNC4	+1	10	+1	7:1:2	−1	3:1	292 ± 7	48.7 ± 0.4	71.7 ± 1.8
UNC5	−1	1	−1	3:1:2	+1	7:1	184 ± 4	61.3 ± 0.7	13.3 ± 0.1
UNC6	+1	10	−1	3:1:2	+1	7:1	494.33 ± 35	60.3 ± 0.3	71.7 ± 1.6
UNC7	−1	1	+1	7:1:2	+1	7:1	218 ± 5	50.1 ± 0.1	35.1 ± 0.1
UNC8	+1	10	+1	7:1:2	+1	7:1	568 ± 29	54.0 ± 0.9	98.2 ± 1.8
UNC9	0	5	0	5:1:2	0	5:1	289 ± 7	52.4 ± 0.6	50.6 ± 0.01

**Table 2:** Coded coefficients, obtained from the factorial regression analysis, evaluating the influence of formulation variables on the average vesicle size of the formulated liposomal models.

Term	Influence on the Average Size						
	Effect	Coef	F-Value	T-Value	*p*-Value	Adj. MS	DF
Constant		296.05		66.60	<0.001		
Lipid concentration	220.66	110.33	615.97	24.82	<0.001	292,141	1
DPPC:DOTAP:CHL	28.24	14.12	10.09	3.18	0.005	4786	1
Lipid phase:Xanthohumol	139.64	69.82	246.69	15.71	<0.001	116,999	1
Lipid conc. × DPPC:DOTAP:CHL	19.26	9.63	4.69	2.17	0.044	2225	1
Lipid conc. × Lipid phase:Xanthohumol	110.06	55.03	153.24	12.38	<0.001	72,677	1
DPPC:DOTAP:CHL × Lipid phase:Xanthohumol	25.64	12.82	8.32	2.88	0.010	3945	1
Lipid conc. × DPPC:DOTAP:CHL × Lipid phase:Xanthohumol	0.66	0.33	0.01	0.07	0.942	3	1
Ct Pt		−6.7	0.25	−0.50	0.624	118	1
Error						474	18

**Table 3:** Coded coefficients, obtained from the factorial regression analysis, evaluating the influence of formulation variables on the ζ-potential of the formulated liposomal models.

Term	Influence on the ζ-Potential						
	Effect	Coef	F-Value	T-Value	*p*-Value	Adj. MS	DF
Constant		53.35		237.58	<0.001		
Lipid concentration	1.47	0.74	10.76	3.28	0.004	13.02	1
DPPC:DOTAP:CHL	−6.49	−3.25	209.09	−14.46	<0.001	252.98	1
Lipid phase:Xanthohumol	6.16	3.08	188.07	13.71	<0.001	227.55	1
Lipid conc. × DPPC:DOTAP:CHL	1.03	0.51	5.23	2.29	0.035	6.32	1
Lipid conc. × Lipid phase:Xanthohumol	−0.06	−0.03	0.02	−0.13	0.898	0.02	1
DPPC:DOTAP:CHL × Lipid phase:Xanthohumol	−2.23	−1.11	24.62	−4.96	<0.001	29.79	1
Lipid conc. × DPPC:DOTAP:CHL × Lipid phase:Xanthohumol	1.45	0.72	10.35	3.22	0.005	12.53	1
Ct Pt		−0.96	2.01	−1.42	0.173	2.43	1
Error						1.21	18

**Table 4:** Coded coefficients, obtained from the factorial regression analysis, evaluating the influence of formulation variables on the entrapment efficiency of the formulated liposomal models.

Term	Influence on the Entrapment Efficiency						
	Effect	Coef	F-Value	T-Value	*p*-Value	Adj. MS	DF
Constant		52.74		51.98	<0.001		
Lipid concentration	46.52	23.26	525.69	22.93	<0.001	12,986.1	1
DPPC:DOTAP:CHL	25.39	12.69	156.57	12.51	<0.001	3867.7	1
Lipid phase:Xanthohumol	3.95	1.97	3.78	1.94	0.068	93.4	1
Lipid conc. × DPPC:DOTAP:CHL	−7.47	−3.73	13.55	−3.68	0.002	334.7	1
Lipid conc. × Lipid phase:Xanthohumol	13.97	6.99	47.43	6.89	<0.001	1171.7	1
DPPC:DOTAP:CHL × Lipid phase:Xanthohumol	−1.5	−0.75	0.55	−0.74	0.470	13.5	1
Lipid conc. × DPPC:DOTAP:CHL × Lipid phase:Xanthohumol	10.08	5.04	24.67	4.97	<0.001	609.5	1
Ct Pt		−2.15	0.5	−0.71	0.489	12.3	1
Error						24.7	18

**Table 5:** Model summary and Analysis of Variance results for the developed models.

Model	S	R-sq	R-sq (adj.)	F-Value	*p*-Value	Adj. MS	DF
Average size	21.78	98.30%	97.54%	129.91	<0.001	61,612	8
Entrapment efficiency	4.97	97.72%	96.71%	96.59	<0.001	2386.1	8
Ζ-potential	1.09	96.16%	94.45%	56.27	<0.001	68.08	8

**Table 6:** Model summary and Analysis of Variance results for models of ζ-potential-based polyelectrolyte titration with iota-carrageenan (CAR) and fucoidan (FUC).

Model	S	R-sq	R-sq (adj.)	F-Value	*p*-Value
CAR	2.88	99.64	99.47	607.77	<0.001
FUC	2.43	99.67	99.51	661.17	<0.001

**Table 7:** L9_3 Taguchi orthogonal array with the formulation variables and corresponding iota-carrageenan (CAR) and fucoidan (FUC) modified models (*n* = 3).

Model	Polysaccharide Concentration		CaCl_2_ Concentration		Incubation Time		Average Size ± SD (nm)		Ζ-Potential ± SD (mV)		PDI ± SD	
	Level	mg/mL	Level	mM	Level	min	CAR	FUC	CAR	FUC	CAR	FUC
CAR/FUC1	1	0.5	1	1	1	30	230 ± 3	121 ± 1	−52.2 ± 0.5	−27.61 ± 0.38	0.30 ± 0.01	0.26 ± 0.01
CAR/FUC2	1	0.5	2	5	2	60	358 ± 3	226 ± 1	−29 ± 2	−25.49 ± 1.01	0.25 ± 0.01	0.17 ± 0.01
CAR/FUC3	1	0.5	3	10	3	120	385 ± 1	391 ± 16	−23 ± 4	−25.45 ± 0.71	0.25 ± 0.02	0.33 ± 0.03
CAR/FUC4	2	1.0	1	1	2	60	335 ± 3	121.6 ± 0.1	−71.48 ± 0.66	−28.94 ± 0.89	0.24 ± 0.01	0.22 ± 0.04
CAR/FUC5	2	1.0	2	5	3	120	374 ± 25	231 ± 1	−30.65 ± 0.24	−26.18 ± 0.06	0.28 ± 0.01	0.14 ± 0.03
CAR/FUC6	2	1.0	3	10	1	30	430 ± 6	430 ± 89	−21.46 ± 0.45	−25.78 ± 0.37	0.53 ± 0.04	0.42 ± 0.03
CAR/FUC7	3	2.0	1	1	3	120	367 ± 2	120.1 ± 0.8	−77 ± 8	−29.63 ± 0.49	0.26 ± 0.01	0.15 ± 0.01
CAR/FUC8	3	2.0	2	5	1	30	504 ± 10	237 ± 3	−29 ± 2	−27.25 ± 0.21	0.53 ± 0.06	0.31 ± 0.02
CAR/FUC9	3	2.0	3	10	2	60	620 ± 59	533.4 ± 42.7	−27.06 ± 0.22	−26.89 ± 0.27	0.66 ± 0.03	0.17 ± 0.03

**Table 8:** Response table for signal-to-noise ratios and analysis of variance.

Variable		Polysaccharide Concentration (mg/mL)			CaCl_2_ Concentration (mM)			Incubation Time (min.)		
Model		Average Size *	ζ-Potential **	PDI *	Average Size *	ζ-Potential **	PDI *	Average Size *	ζ-Potential **	PDI *
CAR	1	−50.01	28.88	11.59	−49.68	36.86	11.56	−51.32	29.04	7.18
	2	−51.55	30.00	9.64	−52.20	26.30	9.54	−52.49	30.73	9.41
	3	−53.75	31.31	6.912	−53.43	27.03	7.05	−51.49	30.42	11.55
	Delta	3.74	2.43	4.67	3.74	10.56	4.51	1.17	1.69	4.37
	Rank	2	2	1	1	1	2	3	3	3
	F-value	84.19	3.25	97.17	75.17	67.22	91.72	1.67	2.78	70.41
	*p*-value	<0.001	0.085	<0.001	<0.001	<0.001	<0.001	0.209	0.109	<0.001
FUC	1	−46.86	28.34	12.18	−41.64	29.16	13.75	−47.33	28.58	9.77
	2	−47.27	28.60	12.58	−47.28	28.39	14.14	−47.78	28.64	14.55
	3	−47.88	28.91	13.87	−53.09	28.31	10.74	−46.90	28.63	14.31
	Delta	1.03	0.57	1.69	11.45	0.85	3.40	0.88	0.05	4.78
	Rank	2	2	3	1	1	2	3	3	1
	F-value	4.99	16.87	1.54	206.18	37.68	8.04	1.01	0.18	7.96
	*p*-value	0.036	<0.001	0.227	<0.001	<0.001	0.009	0.326	0.678	0.010

* smaller is better; ** larger is better.

**Table 9:** R^2^-values of the fitted mathematical models, n.d. = not determined.

Model	Unmodified	Iota-Carrageenan Modified	Fucoidan Modified
Zero-order	0.415	0.729	0.607
First-order	0.910	0.814	0.883
Higuchi	0.671	0.929	0.861
Korsmeyer-Peppas (M_t_/M_∞_ ≤ 0.6)	n.d.	0.964	0.994
Hixson-Crowell	0.783	0.787	0.796
Baker-Lonsdale	0.893	0.919	0.892

## Data Availability

The raw data supporting the conclusions of this article will be made available by the authors on request.

## Supplementary Materials

The following supporting information can be downloaded at: https://www.mdpi.com/article/10.3390/polym18141724/s1, Table S1: Assessment of linearity, limit of detection (LOD), and limit of quantification (LOQ) for the developed HPLC-PDA method for the determination of xanthohumol; Table S2: Assessment of the accuracy of the developed HPLC-PDA method for the determination of xanthohumol; Table S3: Assessment of the precision of the developed HPLC-PDA method for the determination of xanthohumol. Figure S1. Representative HPLC-PDA chromatogram of xanthohumol at a concentration of 20 µg/mL.

## Abbreviations

The following abbreviations are used in this manuscript:

CAR	Iota-carrageenan
CHL	Cholesterol
DMEM	Dulbecco’s Modified Eagle Medium
DOTAP	1,2-Dioleoyloxy-3-trimethylammonium-propane chloride
DPPC	1,2-Dipalmitoyl-sn-glycero-3-phosphocholine
DLS	Dynamic light scattering
EE	Entrapment efficiency
FBS	Fetal bovine serum
FITC	Fluorescein isothiocyanate
FUC	Fucoidan
HFFC	Human foreskin fibroblasts
HPLC-PDA	High-performance liquid chromatography with photodiode array detection
HSD	Honestly significant difference
LD	Limit of detection
LQ	Limit of quantification
PBS	Phosphate-buffered saline
PDI	Polydispersity index
R^2^	Coefficient of determination
S/N	Signal to noise ratio
UNC	Uncoated liposomes
XN	Xanthohumol
