# Supplementary material for: Biopolymer Surface Modification as a Strategy for Conferring “Stealth-like” Characteristics of Xanthohumol-Loaded Liposomes
Source: Polymers (Basel). 2026 Jul 13;18(14):1724. doi: 10.3390/polym18141724 (PMC13417387; doi:10.3390/polym18141724)
Supplement: Supplementary file 1 [file polymers-18-01724-s001.zip › polymers-4379759-supplementary.pdf]

Supplementary materials

Biopolymer surface modification as a strategy for conferring "stealth-like" characteristics of xanthohumol-loaded liposomes

Plamen Simeonov [1,2], Velislava Todorova [2,3], Tsvetelina Batsalova [4], Balik Dzhambazov [4], Stanislava Ivanova [2,3] and Plamen Katsarov [1,2,*]

[1] Department of Pharmaceutical Technology and Biopharmacy, Faculty of Pharmacy, Medical University of Plovdiv, 15A Vasil Aprilov Blvd, 4002 Plovdiv, Bulgaria; plamen.simeonov@mu-plovdiv.bg
[2] Research Institute at Medical University of Plovdiv (RIMU), 4002 Plovdiv, Bulgaria; velislava.todorova@mu-plovdiv.bg (V.T.); stanislava.ivanova@mu-plovdiv.bg (S.I.)
[3] Department of Pharmacognosy and Pharmaceutical Chemistry, Faculty of Pharmacy, Medical University of Plovdiv, 15A Vasil Aprilov Blvd, 4002 Plovdiv, Bulgaria
[4] Department of Developmental Biology, Faculty of Biology, Paisii Hilendarski University of Plovdiv, 24 Tsar Assen Str., 4000 Plovdiv, Bulgaria; tsvetelina@uni-plovdiv.bg (T.B.); balik@uni-plovdiv.bg (B.D.)
* Correspondence: plamen.katsarov@mu-plovdiv.bg

Table S1. Assessment of linearity, limit of detection (LOD), and limit of quantification (LOQ) for the developed HPLC-PDA method for the determination of xanthohumol

Table S2. As-sessment of the accuracy of the developed HPLC-PDA method for the determination of xanthohumol

Table S3. Assessment of the precision of the developed HPLC-PDA method for the determination of xanthohumol.

Figure S1. Representative HPLC-PDA chromatogram of xanthohumol at a concentration of 20 μg/mL.

Table S1. Assessment of linearity, limit of detection (LOD), and limit of quantification (LOQ) for the developed HPLC-PDA method for the determination of xanthohumol

Linear regression	R^2	LOD (μg/mL)	LOQ (μg/mL)
y=91490x+11963	0.9996	1.32	3.99

Table S2. As-sessment of the accuracy of the developed HPLC-PDA method for the determination of xanthohumol

Concentration (μg/mL)	Mean (μg/mL±SD)	Accuracy %	CV%
50	50.07 ± 0.57	100.15	1.13
30	30.29 ± 0.16	100.98	0.53
10	10.05 ± 0.07	100.51	0.16

Table S3. Assessment of the precision of the developed HPLC-PDA method for the determination of xanthohumol.

Concentration (μg/mL)	Intra-day precision			Inter-day precision		
	Mean (μg/mL ±SD)	SEM	CV%	Mean (ng/mL±SD)	SEM	CV%
50	49.85 ± 0.36	0.15	0.72	49.85 ± 0.54	0.22	1.07
30	30.22 ± 0.17	0.07	0.57	30.42 ± 0.32	0.13	1.06
10	10.04 ± 0.03	0.01	0.23	10.06 ± 0.09	0.04	0.90

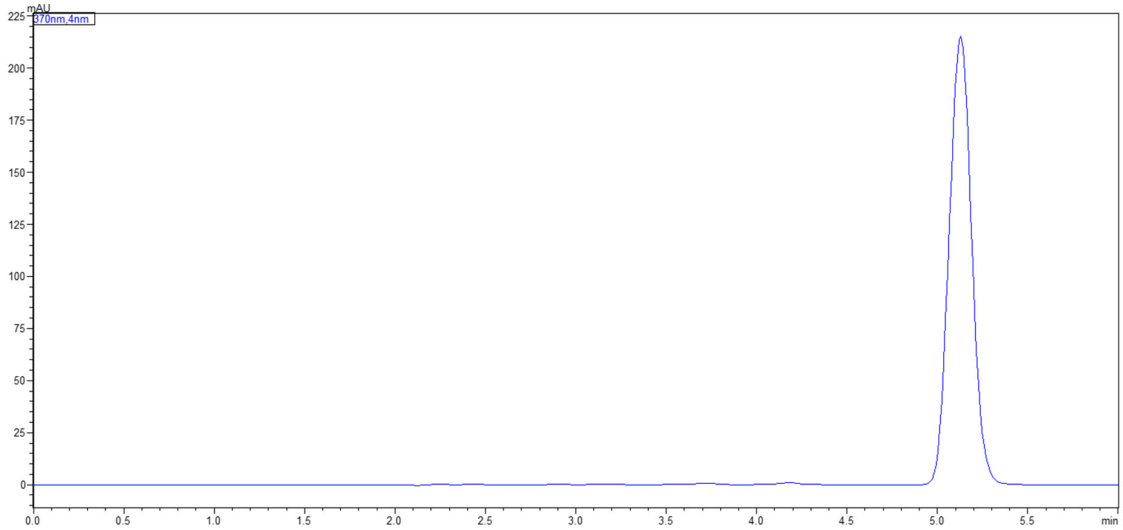

Figure S1. Representative HPLC-PDA chromatogram of xanthohumol at a concentration of 20 μg/mL.
